# *De novo* mutation and somatic mosaicism of gene mutation in type 2A, 2B and 2M VWD

**DOI:** 10.1186/s12959-016-0092-2

**Published:** 2016-10-04

**Authors:** Ming-Ching Shen, Ming Chen, Gwo-Chin Ma, Shun-Ping Chang, Ching-Yeh Lin, Bo-Do Lin, Han-Ni Hsieh

**Affiliations:** 1 Department of Internal Medicine, National Taiwan University Hospital, Taipei, 100 Taiwan; 2 Department of Internal Medicine, Changhua Christian Hospital, Changhua, 500 Taiwan; 3 Department of Genomic Medicine, Changhua Christian Hospital, Changhua, 500 Taiwan; 4 Department of Obstetrics and Gynecology, National Taiwan University Hospital, Taipei, 100 Taiwan; 5 Department of Medical Genetics, National Taiwan University Hospital, Taipei, 100 Taiwan

**Keywords:** Type 2A VWD, Novel mutation, *De novo* mutation, Somatic mosaicism

## Abstract

**Background:**

Von Willebrand disease (VWD) is not uncommon in Taiwan. In type 2 or type 3 VWD hemorrhagic symptoms are severer and laboratory data relatively more distinctive. *De novo* mutation and somatic mosaicism of type 2 VWD gene were rarely reported. Therefore clinical, laboratory and genetic studies of only type 2A, 2B and 2M VWD will be presented and issues of *de novo* mutation and somatic mosaicism will be explored.

**Methods:**

Fifty-four patients belonging to 23 unrelated families from all around the country in whom type 2 VWD exclusive of type 2N has been diagnosed not only by clinical and routine laboratory studies but also by genetic confirmation during 1990–2015 were investigated. A novel technique named amplification refractory mutation system-quantitative polymerase chain reaction (ARMS-qPCR) was used to confirm the presence of somatic mosaicism. Informed consent was obtained for study.

**Results:**

*De novo* mutation was identified in 4 families among 15 families (26.7 %) in whom family members including parents were available for examination. All their parents were free from bleeding symptoms and had no similar mutation as their respective affected daughter.

An interesting example of somatic mosaicism of VWF gene mutation was found in a large family with type 2A VWD. The father carrying a mutated VWF gene, p.Arg1597Trp, transmitted this mutation to his 3 daughters, 1 son, 3 granddaughters and 2 grandsons. However, the father had normal laboratory findings and experienced no abnormal bleeding, while his offspring who inherited the mutation showed abnormal laboratory findings compatible with type 2A VWD and had history of abnormal bleedings. ARMS-qPCR revealed that the father had only 25.5 % mutant in his blood cells and 31.1 % mutant in his oral mucosal cells, while all his offspring had about 49 % mutant in their blood cells.

**Conclusion:**

*De novo* mutation of type 2 VWD gene was identified in 4 out of 15 families (26.7 %) examined. Since only one child was affected in each family, germline mosaicism was not likely. A somatic mosaicism of type 2A VWD gene was documented in a big family by a newly in-house developed technique ARMS-qPCR.

## Background

von Willebrand disease (VWD) is a genetically and clinically heterogeneous inherited hemorrhagic disorder caused by a deficiency or dysfunction of von Willebrand factor (VWF). VWD is considered to be the most common hereditary bleeding disorder [1, 2]. VWD is perhaps not uncommon in Taiwan, however, it has not been well studied. In type 2 and type 3 VWD, hemorrhagic symptoms are severer and laboratory data relatively more distinctive, which lead to diagnose the patient easily [3, 4]. Almost all the mutations detected in type 2 VWD including type 2A, 2B and 2M exhibit autosomal dominant inheritance patterns and is transmitted from either of their parents [3]. However, there was quite few *de novo* mutation of the type 2 VWD gene reported [5], somatic mosaicism of the type 2 VWD gene was even more rarely reported [3], despite it had been mentioned in hemophilia B [6]. Herein clinical, laboratory and genetic studies of only type 2 VWD exclusive of type 2N seen in Taiwan were investigated, which demonstrated a not-so-uncommon occurrence of *de novo* mutation. In this study we cited an interesting example from a big family suffered from type 2A VWD, in which somatic mosaicism of the VWF gene mutation will be presented.

## Methods

Fifty-four patients from 23 unrelated families in whom type 2 VWD has been diagnosed not only by clinical and laboratory studies but also by genetic confirmation during 1990 to 2015 at National Taiwan University Hospital and Changhua Christian Hospital, all tertiary referred hospitals, were investigated. A revised classification of VWD described by Salder JE et al. in 2006 [7] and criteria for the diagnosis of type 2 VWD and its subtypes given by Federici AB in 2014 [8] were used. Factor VIII coagulant activity (VIII:C) was assayed according to the one-stage method. von Willebrand factor antigen (VWF:Ag) was determined by a two-step enzyme immunoassay sandwich method with a final fluorescent detection (ELFA) (VIDAS; bioMérieux, Marcy-I’Étoile, France) [9]. von Willebrand factor activity (VWF:Act) was measured by a latex particle enhanced immunoturbidimetric assay (Instrumentation Laboratory, Lexington, MA, USA). The latex particle was absorbed onto a specific anti-VWF monoclonal antibody directed against the platelet binding site of VWF, i.e. glycoprotein Ib receptor [10, 11]. VWF multimer analysis was performed as described previously [12]. A novel technique named amplification refractory mutation system-quantitative polymerase chain reaction (ARMS-qPCR), which was developed for both preimplantation genetic diagnosis and prenatal genetic diagnosis [13, 14], was used to confirm the presence of somatic mosaicism, because it can accurately quantify the number of copies of a particular amplicon, and determine the ratio of mutant to wild-type alleles in a sample, all using purified DNA as s test sample. Exons and its junction of full length VWF gene were amplified and sequenced as previously described [15]. Informed consent was obtained from patients and family members.

## Results

### Novel mutation in the type 2 VWD

We have seen 54 patients from 23 unrelated families in whom type 2 VWD has been diagnosed during 1990 to 2015, including 35 patients from 11 families of type 2A VWD, 8 patients from 5 families of type 2B VWD and 11 patients from 7 families of type 2M VWD. Type 2N VWD was not found. Among them 19 were male, 35 female; their ages of diagnosis ranged from 1 to 67 years with a median of 24 years. There were no statistical differences in gender and age of diagnosis between various types of VWD.

Eleven different previously reported mutations of the VWF gene based on the ISTH-SSC VWF database, as accessed in June 2016, was identified in the 16 index patients with type 2 VWD including 6 mutations from 8 type 2A VWD patients: p.Arg1374His, p.Ser1506Leu, p.Cys1272Gly, p.Arg1597Trp, p.Cys1272Arg and p.Ile1628Thr; 3 mutations from 5 type 2B VWD patients: p.Arg1306Trp, p.Val1316Met and p.Arg1308Cys; and 2 mutations from 3 type 2M VWD patients: p.Arg1374Cys and p.Arg1315Cys. Six different novel mutations of the VWF gene also according to the ISTH-SSC VWF database was identified in the 7 index patients with type 2 VWD including 3 mutations from 3 type 2A VWD patients: p.Leu1696del, p.Glu1519del and p.Ala1500Val; and 3 mutations from 4 type 2M VWD patients: c.5312-1 ~ −2del AG, p.Leu1276Arg and c.3538 + 1G → C.

### *De novo* mutation in the type 2 VWD

*De novo* mutations of VWF gene were detected in 4 families, one of them was novel. All their parents were free from bleeding symptoms and had no similar mutation as their respective affected daughter. The paternity tests were done in these 4 couples of parents after informed consents were obtained, the kinship were confirmed. The clinical, laboratory and genetic data of these 4 patients were shown in Table 1.

**Table 1:** *De novo* mutations of the VWF gene in the 4 patients with type 2 VWD

Age/Sex	VWD classification	Exon No. (domain)	Nucleotide substitution	Amino acid substitution	VIII:C (IU/dL)	VWF:Ag (IU/dL)	VWF: Act (IU/dL)	Family history and affected sibling in the family
8/F	Type 2A	28 (A2)	c.4517 C → T	p.Ser1506 Leu	53	38.5	10	None
12/F	Type 2B	28 (A1)	c.3916 C → T	p.Arg1306Trp	27	50	7.5	None
10/F	Type 2B	28 (A1)	c.3922 C → T	p.Arg1308Cys	31	35.9	4.5	None
7/F^a^	Type 2M	28 (A1)	c.3827T → G	p.Leu1276Arg^b^	28	26	6	None

^a^F: female

^b^Novel mutation

### Somatic mosaicism of type 2A VWD gene

**Fig. 1:**
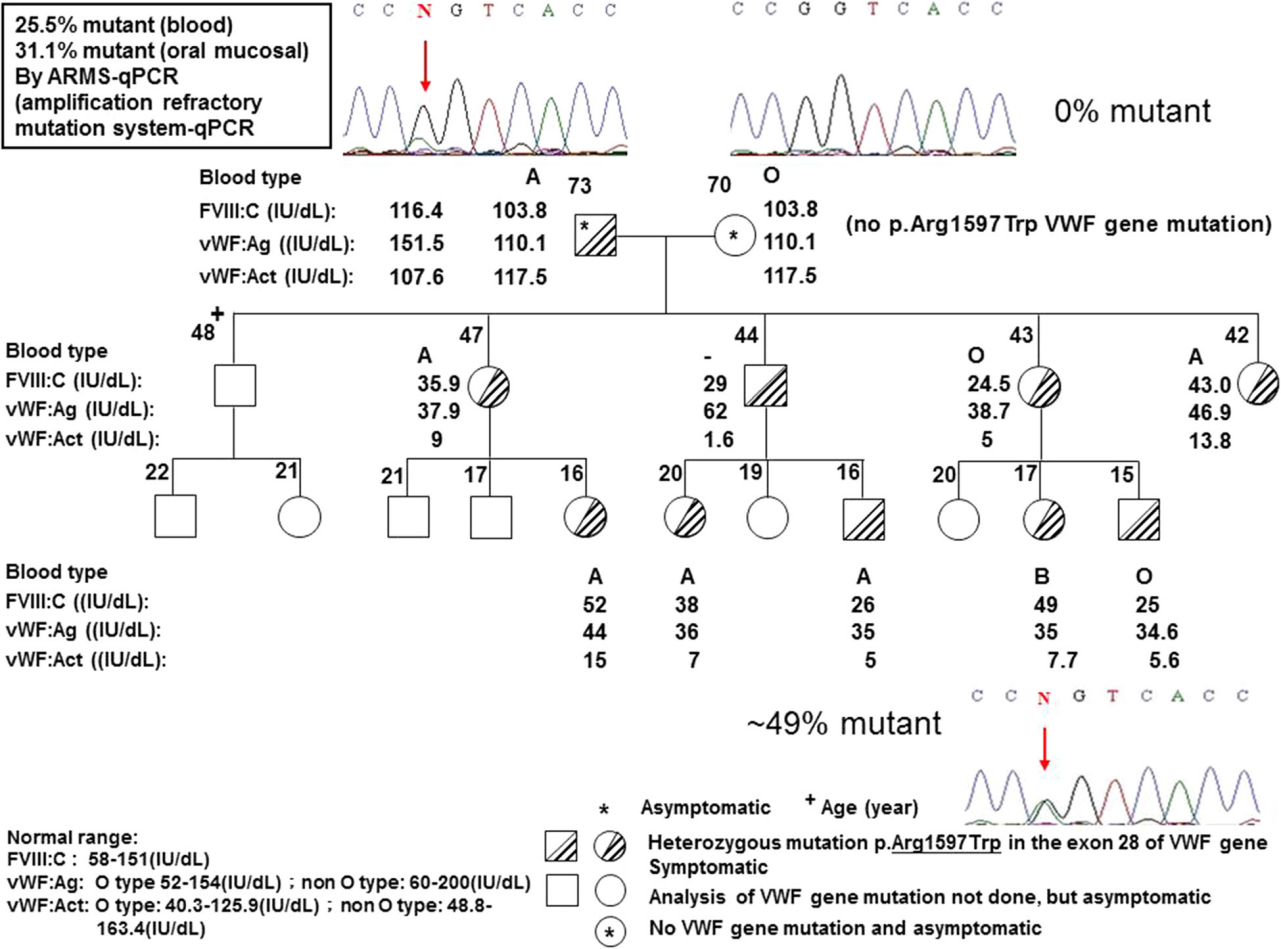
Clinical and laboratory studies as well as amplification refractory mutation system-qPCR(ARMS-qPCR) examination of a family of type 2A VWD with somatic mosaicism of the VWD gene mutation. The father carrying a mutated VWF gene transmitted this mutation to his offspring except the elder son. The father had normal VWF level and experienced no abnormal bleeding but all his offspring who inherited the mutation showed abnormal laboratory findings of type 2A VWD and all had histories of abnormal bleeding. The results of ARMS-qPCR showed that father had only 25.5 % mutant in his blood cells and 31.1 % mutant in his oral mucosal cells. However, all of his offspring had about 49 % mutant in their blood cells. The *arrow* indicates the C/T substitution

**Fig. 2:**
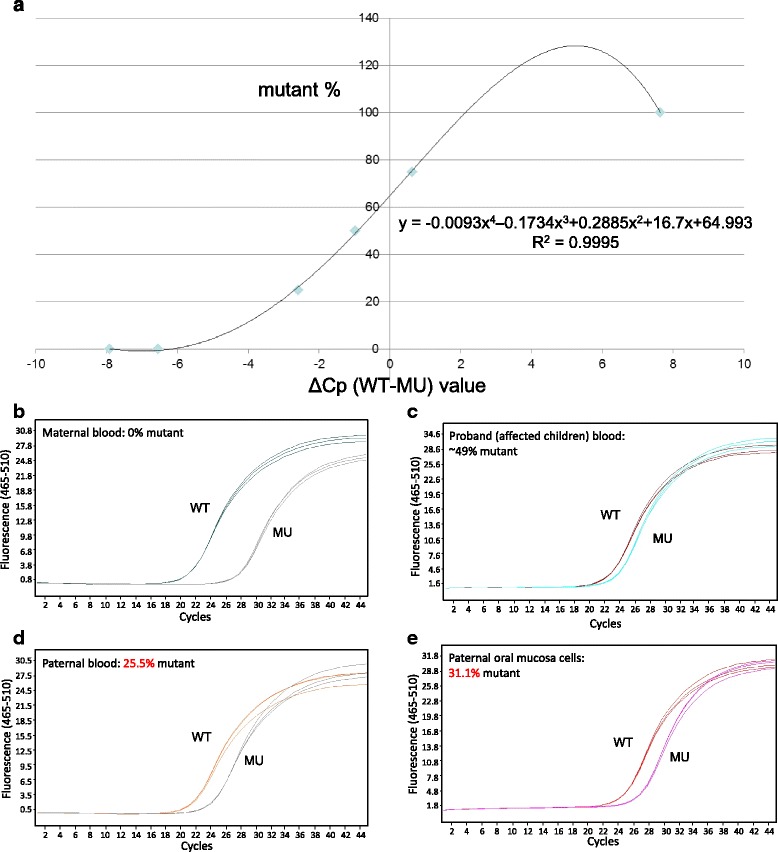
ARMS‐qPCR for the point mutation c.4789C > T (p.Arg1597Trp) of VWF gene. A standard curve was generated by plotting the difference between the PCR cycle crossing point (Cp) of the wild-type (WT) allele and the mutant (MU) allele i.e. ΔCp (WT-MU) value of different synthetic dilutions against the mutant % of the synthetic dilution. The synthetic dilution were prepared by a 2-fold serial dilution of MU DNA by WT DNA. (**a**). According to the S‐curve formula, amplification plot was analyzed at genomic level of an affected family with the paternally‐inherited c.4789C > T mutation to detect the mutant % in different samples of maternal blood (**b**), the proband (affected children) blood (**c**), paternal blood (**d**) and oral mucosal cells (**e**), and the results showed 0 %, 49 %, 25.5 % and 31.1 % in mutant percentage, respectively. Triplicated ARMS‐qPCR tests with WT allele‐specific primers are indicated by WT lines in one color, whereas the tests with MU allele‐specific primers are indicated by MU lines in another color

An interesting example of somatic mosaicism of VWF gene mutation was found in a large family afflicted with type 2A VWD. The father carrying a mutated VWF gene, c.4789C → T, p.Arg1597Trp, transmitted this mutation to his 3 daughters, 1 son, 3 granddaughters and 2 grandsons. However, as shown in Fig. 1, the father had normal levels of FVIII:C, VWF:Ag and VWF:Act and experienced no abnormal bleeding, while his 3 daughters, 1 son and 5 grandchildren who inherited the mutation of VWF gene from the father showed abnormal laboratory findings and VWF multimer analysis (data not shown) compatible with type 2A VWD and all had histories of abnormal bleedings. In order to determine the ratio of mutant (MU) to wild-type (WT) alleles in various tissues from family members, ARMS-qPCR was used. As shown in Fig. 1, the father had only 25.5 % mutant in his blood cells and 31.1 % mutant in his oral mucosal cells, while all his offspring had about 49 % mutant in their blood cells. These findings were compatible to the height of sequence curve of nucleotide C/T as indicated by an arrow in the Fig. 1. The ratio of mutant (%) in each sample as shown above was estimated based on a standard curve generated by plotting the difference between the PCR cycle crossing point (Cp) of the WT allele and the MU allele i.e. ΔCp (WT-MU) value of different synthetic dilutions against the mutant % of the synthetic dilution (Fig. 2a). The synthetic dilutions were prepared by a 2-fold serial dilution of MU DNA by WT DNA. The ARMS-qPCR results for the family afflicted with type 2A VWD were showed in Fig. 2b-e.

## Discussion

Seven out of 23 index patients (30.4 %) or 11out of 54 patients (20.4 %) available for examination showed novel mutations of VWF gene. With a large size of the VWF gene, this high degree of new variant can be expected. Although we consider these normal variants as novel mutations, their pathogenicity for VWD has to be confirmed by linkage analysis or structure-function studies of VWF gene, otherwise some nucleotide changes may be neutral in phenotype and can only be called as a polymorphism. In addition, the pathogenetic basis of some of the changes are also unclear.

*De novo* mutation has been identified in 4 families among 15 families (26.7 %) in whom family members including parents were available for examination. *De novo* mutation is a rare event in VWD [5]. However, some multicenter studies on type 1 VWD had identified *de novo* mutation in at least 2–4 % of proband [16–18]. Such high incidence (26.7 %) of *de novo* mutation in type 2 VWD patients exclusive of type 2N in the present study can be a selection bias or due to small number of patients studied. Two possible explanations for the occurrence of *de novo* mutation are a true event, or results of a new mutation coexisting with a germline mosaicism in a parent, or somatic mosaicism [18]. If more than one child is affected by so called *de novo* mutation with the same mutant, germline mosaicism can be rationally speculated, because the chance of *de novo* mutation with a same genetic defect in two children must be extremely rare [18, 19]. Only one child is affected in each family as found in the 4 families we identified (Table 1), the explanation for this may be a *de novo* mutation or a germline mutation with a transmissible mutant, or somatic mosaicism, unless we are able to obtain a genital tissue for confirmatory analysis. If there is a single affected offspring, germline mosaicism still cannot be excluded, this will depend upon the extent of the mosaic germ cell, a factor that is often impossible to quantify. Germline or gonadal mosaicism has been detected in type 2B VWD [19].

We have found an interesting example of somatic mosaicism of VWF gene in a large family with type 2A VWD with a previously reported VWF gene mutation. There are some key features of autosomal dominant inheritance, i.e., variable expressivity, incomplete or non-penetrance and anticipation. However, there is a general belief that type 2A, 2B and maybe 2M VWD mutations are fully penetrant [3], and are also not associated with variable expressivity. This is in marked contrast with type 1 VWD where all of these genetic variances are common for many mutations, and correlation between genotype and phenotype is difficult [3, 20]. Meanwhile, key features of an autosomal dominant inheritance pattern involves variable expression, incomplete penetrance and anticipation are not associated with type 2 forms of VWD [personal communication with Dr. David Lillicrap].

In this index family of VWD, the father carries the mutant allele but remains asymptomatic throughout his life, while all his children and grandchildren who carry the mutation have abnormal bleeding phenotype, namely patients of VWD. Somatic mosaicism can thus be attributed to this extraordinary presentation of this autosomal dominant disorder since the percentage of the VWF-producing progenitor cells of the vascular endothelium may be very low. From the patients and unaffected mother, the mutant allele versus the wild-type allele follows the classical 50 %:50 % and 0 %:100 %, respectively. However, either the real-time PCR data obtained from the asymptomatic “mutant carrier” were much deviated from 50 %, i.e., 25.5 % in the peripheral blood and 31.1 % in the oral mucosa. This is the undisputed evidence of the existence of “somatic mosaicism”. We therefore speculate that the cell/tissue which are responsible for coding VWF, e.g. vascular endothelial cell or megakaryocyte are carrying the wild-type alleles only, instead of mutant alleles. Such somatic mosaicism have been proved in other disorder such as Dravet syndrome [21 and hemophilia B [6].

## Conclusion

Seven out of 23 index patients (30.4 %) examined showed novel mutations or normal variants of VWF gene. *De novo* mutation of type 2 VWD gene was identified in 4 families among 15 families (26.7 %) in whom family members were available for examination. Only one child was affected in each family. Germline mosaicism is therefore unlikely although its possibility cannot be totally excluded. Somatic mosaicism of VWF gene mutation was documented in a large family with type 2A VWD with a previously reported VWF gene pathogenic mutation, p.Arg 1597Trp. This extraordinary presentation was confirmed by a newly developed technique named ARMS qPCR.

## Abbreviations

VWD: von Willebrand disease
VWF: von Willebrand factor
ARMS-qPCR: Amplification refractory mutation system-quantitative polymerase chain reaction
